# Splitting Tensile Strength of Cement Soil Reinforced with Basalt Fibers

**DOI:** 10.3390/ma13143110

**Published:** 2020-07-12

**Authors:** Shengnian Wang, Fangyuan Chen, Qinpei Xue, Peng Zhang

**Affiliations:** College of Transportation Science & Engineering, Nanjing Tech University, Nanjing 211816, China; shengnian.wang@njtech.edu.cn (S.W.); 662081803003@njtech.edu.cn (F.C.); xueqinpeii@163.com (Q.X.)

**Keywords:** cement soil, basalt fiber, mixing procedure, splitting tensile strength, toughening mechanism

## Abstract

Due to low splitting tensile strength, cement soil is more likely to experience dry shrinkage and cracking in practical engineering. In this study, the mixing procedure of the cement soil reinforced with basalt fibers was investigated; the influences of cement content, curing time, basalt fiber content and length on the splitting tensile strength of the cement soil reinforced with basalt fibers were studied; and the correlation of the splitting tensile strength vs. the compressive strength of the cement soil reinforced with basalt fibers was discussed. The contribution of basalt fibers on performance improvement of the cement soil was also addressed based on the microstructural analysis and the toughening mechanism exposition. Results indicate that the best mixing method for the cement soil reinforced with basalt fibers is to mix the muddy silty clay with basalt fibers first, then with cement slurry. The increase of cement content and curing time can improve the splitting tensile strength of the cement soil effectively. The splitting tensile strength of the cement soil increases first and then decreases with the content and length of basalt fibers. The optimal content and length of basalt fibers for the cement soil are 0.4% and 12 mm, respectively. The relationship between the splitting tensile strength and the compressive strength of the cement soil reinforced with basalt fibers can be described as a linear relationship with the correlation coefficient of 0.245 and the determination coefficient of 0.990. The contribution of basalt fibers on the toughening mechanisms of cement soil shows that the fiber-matrix interaction would be the dominant effect to control the tensile strength of the soil-cement-fiber composites. The results of this study can provide a reference for the design and application of cement soil reinforced with basalt fibers in actual engineering.

## 1. Introduction

Due to its flexible reinforcement style, great water-sealing effect, little construction disturbance, and low cost, cement soil has been widely used in the reinforcement of soft soil foundations, core walls of earth-rockfill dams, and other projects [1]. However, the low tensile strength of the cement soil always leads to the cracking of the supporting structure, and hydraulic fracturing failure in the core wall of the earth-rockfill dam. How to improve the tensile strength of the cement soil has thus been a hot issue discussed by domestic and overseas scholars and engineers up to now [2,3].

Previous research had shown that the internal structure of the cement soil would be changed when the cement content was higher than 10% [1,4,5]. The ultimate bearing capacity and the strain corresponding to the failure load of the cement soil would be much higher than the plain soil when the cement content continued to increase. However, it is not the case that greater cement content will lead to better ultimate bearing capacity of the cement soil. When the cement content is exorbitantly high, the excessive volume shrinkage caused by evaporation of water during the hardening process will result in cracking of the cement soil. In other words, the failure of the cement soil with high cement content will change into brittle fracture and tensile failure from plastic failure gradually [6,7]. Low tensile strength is thus the fatal flaw of the cement soil.

Many scholars and engineers have already made improvements and innovations for improving the performance of the cement soil, such as adding soil stabilizers, mineral admixtures, and other additives [8,9,10,11,12]. Basalt fiber is a kind of filamentous mineral admixture made from natural basalt ore at high temperatures. It has much higher tensile strength, deformation modulus and corrosion resistance than other kinds of common fiber admixture. Moreover, it has the same mineral composition as the basalt ore, and can degrade into the soil directly. Hence, the basalt fiber has been regarded as a natural green and environment-friendly mineral admixture in the 21st century [13,14,15]. For example, many research works have presented experimental investigations of the mechano-physico-chemical properties of basalt fiber reinforced concrete or polymer composites affected by the volume fraction of chopped basalt fibers and discussed the computational formulas for the evaluation of the tensile strength of fiber-reinforced materials [16,17,18,19,20,21]. All these documented research works indicate that the basalt fiber is cost-effective and offers exceptional properties over glass fibers. Therefore, adding basalt fiber into the cement soil will certainly enhance the integrity and bearing capacity of the cement soil, solving the problems of brittleness and poor ductility. Moreover, it will also improve the cement soil’s static-dynamic strength performance, dry shrinkage cracking resistance, and impermeability under high water pressure effectively. However, there are few studies on the application of basalt fiber in geotechnical engineering, and no systematic studies on the mechanical properties of the cement soil reinforced with basalt fiber up to now [22,23].

In this study, we will make a thorough exploration on the splitting tensile strength of the cement soil reinforced with basalt fiber by plenty of diametric compression tests, and discuss the impacts of cement content, curing time, basalt fiber content and length on the tensile strength of the cement soil reinforced with basalt fiber. The results of this study are expected to provide experimental evidence for the application of the cement soil reinforced with basalt fiber in practical engineering.

## 2. Materials and Methods

### 2.1. Materials

#### 2.1.1. Soil

The muddy silty clay used in this study was derived from a construction site at the Ligang community, Jiangyin, China. The physical indexes of this soil are shown in Table 1. According to the interrelated relationship among the void ratio *e*, water content *w*, specific gravity *G*_s_, and saturation degree *S*_r_ as *e* = *wG_s_/S_r_*, this soil with the void ratio greater than 1.0 should be a kind of highly compressible clay.

#### 2.1.2. Basalt Fiber

**Figure 1:**
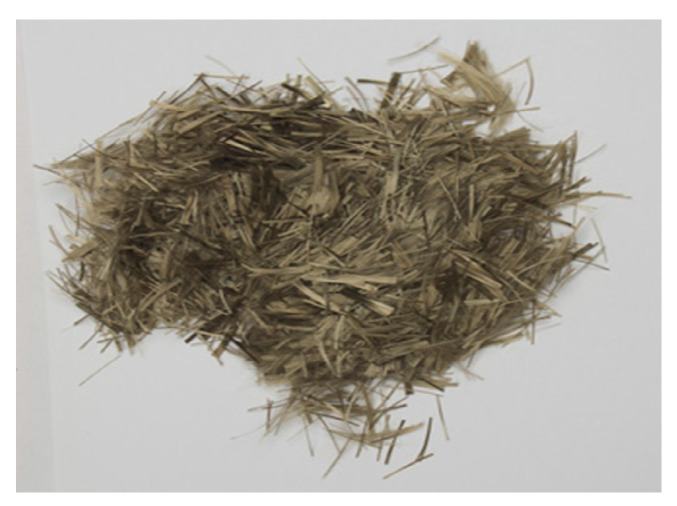
Alkali-resistant basalt fiber.

Studies on corrosion resistance of basalt fiber in an alkali environment have been widely carried out by domestic and overseas scholars. However, the conclusions of these studies on the alkali resistance of basalt fiber are inconsistent. Some of them pointed out that the basalt fiber has better alkali-resistance than carbon fiber and glass fiber at room temperature [24,25]; by contrast, others indicated that the mass loss rate of basalt fiber in a strong alkaline solution was higher than 40% and may be responsible for the loss of later strength [26,27,28]. To avoid this inconsistency, the fiber used in this study is an alkali-resistant basalt fiber, which is manufactured by Jiaxing Anlide Construction Engineering Co., Ltd. (Jiaxing, China), and is composed of basalt fiber bundles with metallic colors, as shown in Figure 1. The acid and alkali resistance of this alkali-resistant basalt fiber is greater than 99%, and can ensure that it has a good stability in the alkali environment formed by cement hydration. The performance parameters of this alkali-resistant basalt fiber are as shown in Table 2.

#### 2.1.3. Cement

Composite Portland cement (P.C 32.5) is used as the cementing agent. The specific gravity was 3.15. The specific surface was greater than 300 m^2^/kg. The initial setting time was greater than 45 min. The compressive strengths after 3 days and 28 days were 10 MPa and 32.5 MPa, respectively.

### 2.2. Sample Preparation

**Figure 2:**
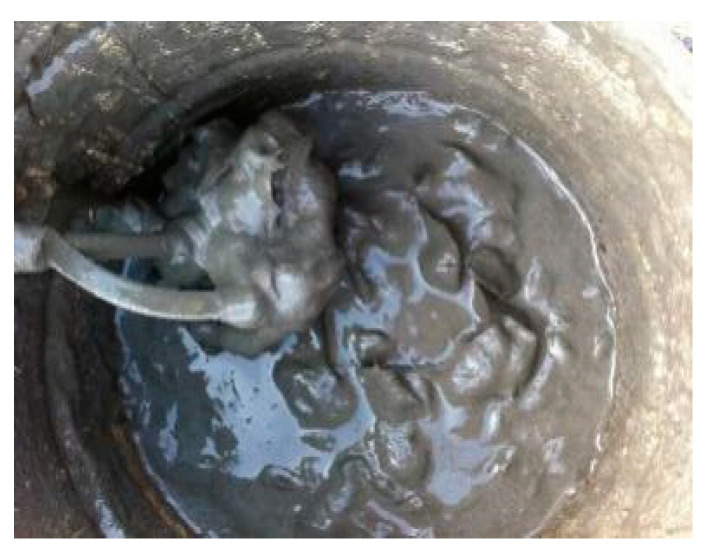
Cement paste mixed basalt fiber directly.

**Figure 3:**
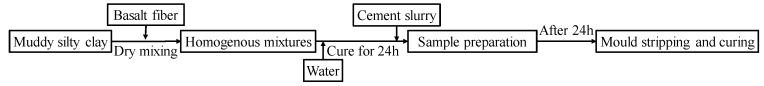
The mixing procedure of cement soil reinforced with basalt fiber.

The key to making cement soil samples is to disperse the basalt fibers into the cement soil evenly. Unfortunately, laboratory tests have indicated that if the basalt fiber bundles were mixed and stirred with the cement soil slurry directly, the basalt fibers would tend to experience uneven dispersion and form clumps or balls, as shown in Figure 2, and even very flowable matrices might not pass through the congested fiber network properly. This, however, would lead to great difficulties in actual application. Here, we find a new mixing procedure [29]. The core step is to mix the basalt fiber bundles with sodium bentonite as a mass ratio of 20:1 fully first, blend with dry muddy silty clay, and then stir with the cement slurry thoroughly. The first step can drive the basalt fiber bundles dispersed fully and can avoid the basalt fiber broken during the mixing, the second step can make the basalt fiber distributed in muddy silty clay evenly, and the third step can be convenient for ongoing fieldwork and quality control. The specific mixing procedure is shown in Figure 3.

(a) Air-dry the on-site sampled muddy silty clay first and then pulverizes.
(b) Filter off the impurities by a sieve with an aperture of 2 mm.
(c) Determine the water content of the air-dried muddy silty clay.
(d) Mix basalt fibers with sodium bentonite as a mass ratio of 20:1 first and make sure that the basalt fibers are dispersed fully.
(e) Mix basalt fibers with the air-dried fine muddy silty clay according to the specified mixture design, and stir well to ensure all basalt fibers are dispersed in muddy silty clay evenly. In this study, the homogeneity of fiber distribution in the air-dried fine muddy silty clay was guaranteed by the mixing time, which was determined by trial and error.
(f) Determine the water consumption for the air-dried fine muddy silty clay mixed with basalt fibers based on the water content of the on-site muddy silty clay (36%).
(g) Add the water into the air-dried fine muddy silty clay mixed with basalt fibers, blend fully, and cure for 24 h in a hermetically sealed container to eliminate the impact of clay hydration.
(h) Prepare cement slurry with the water:cement ratio of 0.5, and fill it into the dry fiber-soil mixture at small increments and fully stir.
(i) Remold the samples with sizes of 50 mm in diameter and 100 mm in height, in which the fully mixed fiber-cement-soil mixture is divided into four parts evenly first and then are carefully pouring into the mold in turns. When the soil sample is compacted to the specified height each time, the surface of the soil sample is roughened before pouring another part of the mixture into the mold. The purpose of this treatment is to eliminate the weak contact between adjacent layers.
(j) Removal the mold after standing for 24 h.
(k) Weigh the quantity of the prepared samples, and if the quality of samples has deviated from the mean value by 20%, those samples will be discarded. The main purpose of this step is to eliminate the difference in sample preparation. In this study, each mixing ratio was prepared with six samples at least, but only six samples with the best quality will be selected and further cured under three curing times, including 7 days, 28 days, and 90 days. The relative humidity of standard curing is greater than 95% and the temperature range is 20 ± 3 °C.
(l) Considering that this study pays more attention to saturation strength, the prepared samples were cured in water for 24 h before the expiration of the curing time, and then mechanical testing was carried out.

### 2.3. Testing Method

**Figure 4:**
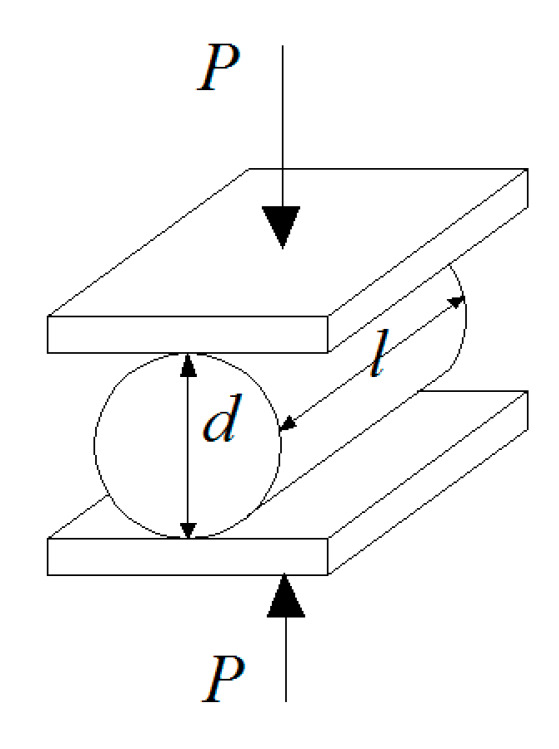
Diagram of diametric compression test loading.

The splitting tensile strength of the cement soil reinforced with basalt fiber in this study was gained by the diametric compression test indirectly [30]. As shown in Figure 4, the computational formula of splitting tensile strength based on the linear elastic theory is defined as follows.

(1) $$\sigma_{t}=\frac{2P}{\pi dl}$$

where *σ_t_* is the tensile strength of the cement soil, MPa; *P* is the failure load, kN; *d* is the diameter of the sample, mm; *L* is the length of the sample, mm.

In this study, the split line on the sample surface is determined firstly when the diametric compression test is required, and then the sample is placed in the center of the pressure plate, keeping the split line of the sample consistent with the centerline of the pressure plate at the bottom. The sample will be continuously loaded at 8 mm/min until the load reaches its peak value and then dropped rapidly.

### 2.4. Testing Plan

The production and curing of the cement soil in this study shall be carried out strictly by the specification for mix proportion design of cement soil (JGJT2332011), issued by China [31]. The experiment schemes are designed according to cement content, curing time, basalt fiber content (the percentage of basalt fiber in the mass of air-dried muddy silty clay) and length, as shown in Table 3, in which the cement is referred to as C and the basalt fiber is referred to as B. The reason why the cement content 15% was adopted for the analyses of basalt fiber content and length here is attributed to two aspects: the one is that the mixing ratio of cement in the soil is generally 7–15% for the engineering application of cement soil in China, the other is that the upper value of cement content (15%) can almost meet the improvement requirement of most soils and the engineering economy in practice.

## 3. Results and Discussion

### 3.1. Splitting Tensile Strength and Failure Characteristics

The splitting tensile strength of the samples obtained from the peak load of the diametric compression test are shown in Table 4, in which *C*_v_ is the variation coefficient. It can be found that the mean tensile strengths have relatively low variation coefficients so that the following discussion on the significance of influence factors will be carried on by observation and statistical fitting.

**Figure 5:**
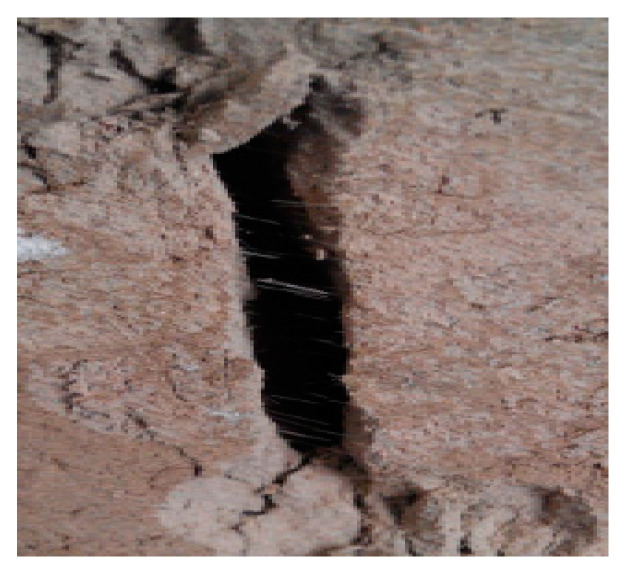
Failure characteristic of cement soil reinforced with basalt fiber.

Figure 5 shows the failure characteristics of the cement soil reinforced with basalt fibers in the diametric compression test. It can be seen that there is a large amount of basalt fibers distributed in the crack of the sample. The inclusion of basalt fibers and consequently enhancing the bridging effect should play a very important role in the initiation and extension of cracks, thereby affecting the overall performance of the soil-cement-fiber composites [21]. Although the high basalt fiber content may result in a porous structure and heterogeneous fiber-matrix interaction, the strong interfacial bond is achieved due to the hydrophilic characteristics and rough furrows at the surface of basalt fibers, forming an annular region surrounding the basalt fibers, and thus results in tensile stress induced by the compressive load transformed into shear stress at the fiber-matrix interface and resisted through the adhesion and friction at their contact surfaces. Moreover, the basalt fibers will alleviate the internal concentrated stresses applied in different orientations by transferring them into a uniform distribution, thereby avoiding stress concentration and local fractures. The considerably higher tensile strength of the basalt fibers demands that they should be dragged under crack bridging or pulled out before cracks propagate by an higher energy of fracture. Therefore, the basalt fibers in the cement soil do have a resistance effect on the splitting failure of the cement soil.

### 3.2. Influence of Cement Content on Maximum Tensile Strength

**Figure 6:**
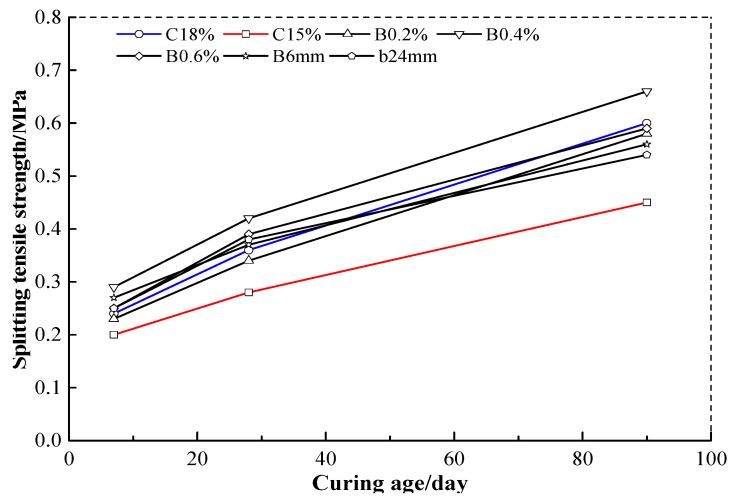
The splitting tensile strength changes of cement soil with different mixture design.

Figure 6 shows the splitting tensile strength changes of cement soil with different mixture design. The contrast of the C15% and the C18% shows that the splitting tensile strength of C18% is 20.0%, 28.6%, and 33.3% higher than that of C15% when the curing times are 7 days, 28 days and 90 days, respectively. This outcome obviously is consistent with general cognition. However, it is also well known that the more cement content will not always lead to higher strength performance and economic efficiency, especially when the cement content is much higher. The fiber has been used for improving the mechanical properties of the cement soil for many years. Previous studies had pointed out that whether increasing cement content or mixing basalt fiber will improve the splitting tensile strength of the cement soil [32,33,34]. The results of this study show that the splitting tensile strength of the cement soil reinforced with basalt fiber is indeed always greater than that of plain cement soil, and this difference will be enlarged with the development of curing time. In particular, when the content and length of basalt fiber are 0.4% and 12 mm, the splitting tensile strengths of the cement soil (cement content 15%) reinforced with basalt fiber at the curing time of 90 days will increase by 10% compared with the plain cement soil with cement content 18%. This indicates that the tensile performance of the cement soil can be improved significantly by adding a certain content of basalt fiber. In other words, the improvement of cement content on the tensile strength of soil mass can be replaced by basalt fiber’s reinforcement effect. The main reason should be that the basalt fibers with considerably higher tensile strength can still contribute to resistance to cracking when the plain cement soil fails and, therefore, improves the tensile properties of the cement soil effectively.

### 3.3. Influence of Curing Time on Maximum Tensile Strength

As shown in Figure 6, the average growth rate of the splitting tensile strength of the cement soil is roughly similar when the curing times are 7 days, 28 days, or 90 days, and the long curing time will lead to the better tensile strength for the cement soil. Several studies had pointed out that there seems to be an approximate positive correlation among splitting tensile strengths of the cement soil reinforced with fiber at different curing times [35,36]. Therefore, a linear relationship between the early and later strengths of the cement soil reinforced with or without basalt fibers is considered. Here, to keep things simple, the splitting tensile strength of the cement soil at both the curing times of 28 days and 90 days is evaluated approximately taking the case of 7 days for reference.

(2) $$\sigma_{t28}=\left(1.37\sim 1.56\right)\sigma_{t7}$$

(3) $$\sigma_{t90}=\left(1.95\sim 2.65\right)\sigma_{t7}$$

where σ_t7_, σ_t28_, and σ_t90_ refer to the splitting tensile strength of the cement soil with the curing times of 7 days, 28 days, and 90 days, respectively.

### 3.4. Influence of Fiber Content on Maximum Tensile Strength

**Figure 7:**
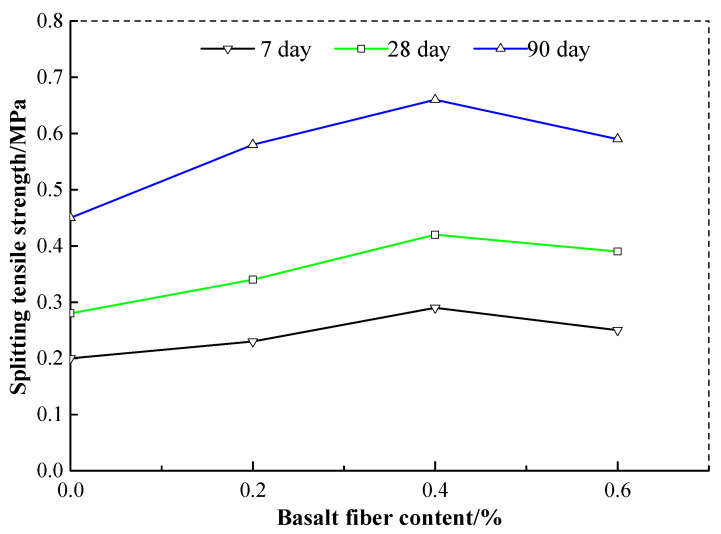
The relations between basalt fiber content and splitting tensile strength.

Figure 7 shows the splitting tensile strength curves of the cement soil with basalt fiber content of 0.2%, 0.4%, and 0.6%. Here, the cement content is 15%, and the basalt fiber length is 12 mm. It can be found that the splitting tensile strength of the cement soil is increasing first and then decreasing with the basalt fiber content. So the optimal basalt fiber content mixed in the cement soil should be 0.4%. The reason for the splitting tensile strength increasing first and then decreasing may be explained by the fact that excessive basalt fiber bundles distributed parallel to the splitting surface result in more weak surfaces and insufficient contacts among soil particles [33,37]. The higher the basalt fiber content is, the greater the risk of the failure along these weak surfaces will be.

Table 5 shows the growth ratio of splitting tensile strength of the cement soil with different basalt fiber content. It illustrates that the splitting tensile strength of the cement soil reinforced with basalt fibers is always higher than that of the plain cement soil with cement content 15%. The cement soil with basalt fiber content 0.4% has the highest growth ratio at a curing time of 28 days but a second growth ratio at a curing time of 90 days. The reason for this difference may be that the development of fiber-matrix interaction is a time-dependent mechanism. The strength of the matrix and its interfacial bond with the basalt fibers is increased over time, especially at early ages. However, the enrichment productions of cement hydration (Ca(OH)_2_) may cause an absolute volume shrinkage through the cementitious matrix, resulting in considerable microcracks. The high capillary pressure produced between wet and dry zones of the micropore network during the hardening of the cement soil reinforced with basalt fibers may further aggravate the development of these dry shrinkage cracks. Hence, the low growth ratio of the later strength of the cement soil reinforced with basalt fibers arises.

### 3.5. Influence of Fiber Length on Maximum Tensile Strength

**Figure 8:**
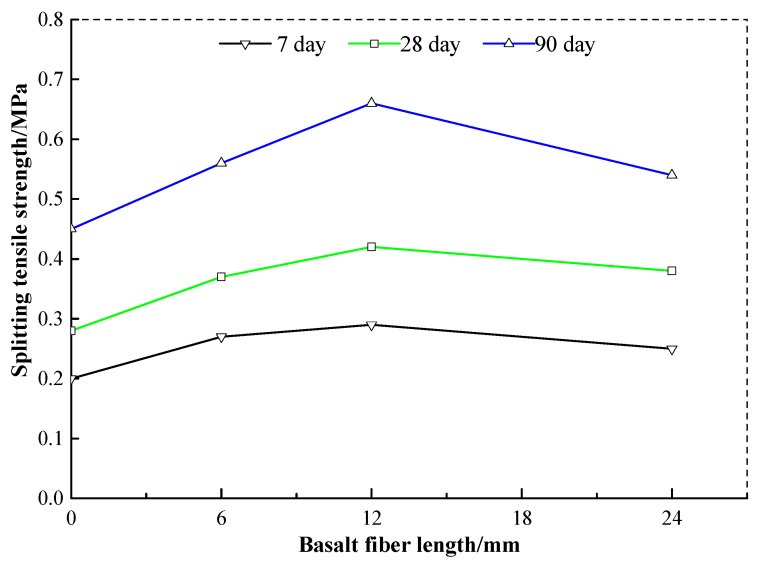
The relations between basalt fiber length and splitting tensile strength.

Several studies have proved that the fiber addition will help in achieving a great development in the strength of soils improved with lower cement content, and the impact of fiber length on the strength of the cement soils cannot be overlooked [5,34,38]. If longer basalt fiber is used, the mixing of the fiber and the cement soil would be very difficult and cause the distribution of the fiber in the cement soil to be extremely uneven. If shorter basalt fiber is used, the basalt fiber would be pulled out easily under the load, and could not play the role of reinforcement on the cement soil [21,39,40]. Therefore, the basalt fiber length in the cement soil must be moderate. Figure 8 shows the splitting tensile strength curves of the cement soil with basalt fiber length of 6 mm, 12 mm, and 24 mm. Here, the cement content is 15%, and the basalt fiber content is 0.4%. The splitting tensile strength of the cement soil with the same basalt fiber content and curing time shows that there is a trend of first increasing and then decreasing. So the optimal basalt fiber length mixed in the cement soil should be 12 mm.

Table 6 shows the growth ratio of splitting tensile strength of the cement soil reinforced with basalt fiber. It illustrates that the splitting tensile strength of the cement soil reinforced with basalt fiber is always higher than that of the plain cement soil with cement content of 15%. The cement soil with basalt fiber length of 12 mm has the highest growth ratio at a curing time of 28 days, which also proves the conclusion on the optimal basalt fiber length of the cement soil.

## 4. Correlation of Splitting Tensile Strength vs. Compressive Strength

**Figure 9:**
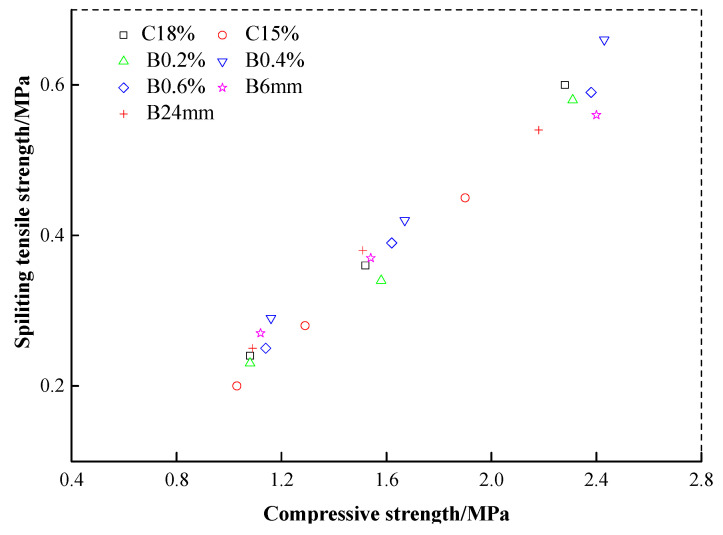
The relationship between compressive strength and tensile strength.

Several studies have pointed out that there was a linear relationship between the tensile strength and the compressive strength of the cement soil [32,33]. Therefore, the correlation between the splitting tensile strength and the compressive strength of the cement soil reinforced with basalt fibers was investigated in this study. Table 7 illustrates the unconfined compressive strength of the samples with the same testing plan, preparation method, and curing conditions of the tensile strength test, in which the mean compressive strengths also have relatively low variation coefficients. Figure 9 shows a scatter diagram of the splitting tensile strength vs. compressive strength based on the experimental data for both fiber-cement stabilized soil and cement soil with variations of cement content, curing time, fiber content and length. As can be seen in this figure, the splitting tensile strengths of the cement soil and the fiber-cement stabilized soil are increasing with the compressive strength. Strong linear relationships can be found between them.

Table 8 presents the fitting relationships between the splitting tensile strength *σ*_t_ and the compressive strength *σ*_c_ of the cement soil and the fiber-cement stabilized soil, in which these linear relationships are fitted by the function as shown in Equation (4).

(4) $$\sigma_{t}=a\sigma_{c}$$

where *a* is the correlation coefficient.

It can be found that strong linear relationships between the splitting tensile strength and the compressive strength of the cement soil with or without fiber inclusion were achieved with the high determination coefficient (R^2^ = 0.982 for the cement soil and R^2^ = 0.990 for the fiber-cement stabilized soil). The general relationships between the splitting tensile strength and the compressive strength for the cement soil with and without fiber inclusion are:

(5) $$\{\begin{array}{c} \sigma_{t}=0.240\sigma_{c}\\ \sigma_{t}=0.245\sigma_{c} \end{array}\begin{array}{c} ,\\ , \end{array}\begin{array}{c} R^{2}=0.982\\ R^{2}=0.990 \end{array}\begin{array}{c} \;\mathrm{for}\;\mathrm{cement}\;\mathrm{soil}\;\mathrm{without}\;\mathrm{fiber}\;\mathrm{inclusion}\;\\ \mathrm{for}\;\mathrm{cement}\;\mathrm{soil}\;\mathrm{with}\;\mathrm{fiber}\;\mathrm{inclusion}\; \end{array}$$

As can be seen in the Equation (5), the cement soil reinforced with basalt fiber obviously has a better correlation of the splitting tensile strength and the compressive strength, and a better improvement in splitting tensile strength with the correlation coefficient of 0.245.

## 5. Improvement Contribution of Basalt Fibers

### 5.1. Microstructural Characterization

**Figure 10:**
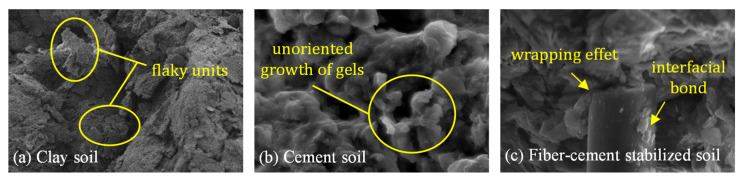
Typical microstructure scanning electron micrograph (SEM): (**a**) clay soil; (**b**) cement soil; (**c**) fiber-cement stabilized soil.

To evaluate the stabilization effect of the soil stabilized with cement and basalt fibers, microstructure observations by scanning electron microscopy (SEM) (JSM-5900, Nanjing Tech University, Nanjing, China) were carried out for the plain clay soil, and the cement soil with and without fiber inclusion, in which the cement content was 15%, and the fiber content and length were 0.4% and 12 mm, respectively. Figure 10 illustrates the typical microstructures of these three kinds of soils at magnifications of 4000×. It can be found that the plain clay soil is mainly composed of flaky units as shown in Figure 10a. The links among flaky units are the main contact, but does not exclude cementitious connections. The structure of the plain soil mainly consists of particle-aggregate, contact-cementation, and micro-trellis pores. The distribution of particles in the composite Portland cement soil as shown in Figure 10b is clustered and has an obvious characteristic of the unoriented growth of gels in space. The hydration of the cement, on the one hand, promotes the soil particles bonded together, leading to the agglomerated particle size of the composite Portland cement soil increased (not excluding the existence of local overhead between large particles); on the other hand, it also results in a strong compacted granular-inlaid-cementitious structure so that the higher macroscopic strength performance is achieved. The microstructure of the cement soil reinforced with basalt fibers as shown in Figure 10c illustrates that the basalt fiber having a rough surface is tightly wrapped by the soil and the hydration products, presenting a better interfacial bond between the cement soil and the basalt fiber. This interfacial bond effects increase the interaction between the cement soil and the basalt fiber, and thus can explain the reason why the fiber improves the strength of the cement soil.

### 5.2. Toughening Mechanisms

Shrinkage of cement soil can be categorized into chemical, autogenous, and dry shrinkages. When the evaporation rate of water near the surface of the cement soil is greater than its bleeding rate, the chemical shrinkage highly correlated to the setting time of the cement will result in an absolute volume change [41,42]. The autogenous shrinkage of the cement soil is always caused by negative capillary pressure due to the water consumption of cement hydration. The dry shrinkage of the cement soil is the result of irreversible shrinkage caused by further water loss, which will lead to a micropore network, and might even cause microcrack propagation. In general, both the interface frictional effect and the spatial confined effect are responsible for the tensile strength growth of the fiber-reinforced soil [43]. However, once the binder is used, the fiber-matrix interaction would be the dominant effect to control the overall mechanical properties of the soil-cement-fiber composites [21]. The strong fiber-matrix interaction and the higher Young’s modulus of the fiber may also result in controlling shrinkage and enhancement of the mechanical properties of the soil-cement-fiber composites [44]. The contribution of basalt fibers on the toughening mechanisms of cement soil can be explained by the following aspects.

(i) Strong interfacial bond. The basalt fibers with hydrophilic characteristics and rough furrows at the surface will result in firm contact with the matrix (the cement soil), and this contact will be continuously strengthened by binder development. Meanwhile, an annular region surrounding the basalt fibers will be formed over time by the dual action of fiber and matrix, thereby further enhancing this interfacial bond. Once this interfacial bond is strong enough to limit the subsequent shrinkage, the trapped matrix between the fibers will be confined, leading to a higher load-bearing capacity. This strong interfacial bond will also cause tensile stress in failure zones transformed into shear stress at the fiber-matrix interface and resisted through the adhesion and friction at their contact surfaces when the cement soil reinforced with basalt fibers is subjected to an external load.
(ii) Effective bridging effect. In general, increasing the basalt fiber content and consequently enhancing the bridging effect can improve the integrity of cement soil effectively, and alleviate the internal concentrated stresses applied in different orientations by transferring them into a uniform distribution. This mechanism implies that a large number of fibers bridging over an extended length can significantly contribute to the amount of energy absorption of the soil-cement-fiber composites. Besides, although increasing the fiber content will also result in poor compaction, and the formation of many microcracks instead of a few large macrocracks, the cracks are initiating locally where the applied stress is higher than the tensile strength of the matrix. Once a crack faces fibers, those bridging fibers will sustain and transfer stresses by the deformation of the fibers itself and the friction between the fibers and the matrix. The bridging effect at a crack will be lost when the bridging fibers are pulled out or broken. This, however, will demand much higher external work, and result in the formation of multiple microcracks and pseudo strain hardening.
(iii) Crack deflection and branching effect. When a crack reaches to fibers, the fibers may debond due to insufficient fiber length or interfacial bond. The interaction of propagating micro-cracks and the position of the other fibers may result in the crack deflecting or branching in-plane. In particular, when the fibers with higher Young’s modulus are used, the crack-propagation behaviors such as deflection and branching will prevent the tendency of crack localization along a certain path, and thus increase the effectual crack path to cause energy absorption and enhance the toughness.

## 6. Conclusions

The low tensile strength of the cement soil always limits its engineering application. In this study, splitting tensile test, compressive tests, and SEM tests were conducted to study the influences of cement content, curing time, basalt fiber content and length on the splitting tensile strength of the cement soil reinforced with basalt fibers and the improvement contribution of basalt fibers on strength performance. Some main conclusions are obtained as follows:

(1) The key to making cement soil samples is to distribute the basalt fibers into the cement soil evenly. The best way to solve the difficulties in engineering mixing and facilitate ongoing fieldwork and quality control is to mix the muddy silty clay with basalt fibers first, then with cement slurry.
(2) The improvement of cement content on the strength of soil mass can be replaced by basalt fiber’s reinforcement effect. However, the greater basalt fiber content and longer basalt fiber length do not always lead to a better reinforcement effect for the cement soil. The ideal reinforcement effect of basalt fiber for the cement muddy silty clay will be achieved when the basalt fiber content and length are 0.4% and 12 mm.
(3) The strong linear relationships between the splitting tensile strength and the compressive strength of the cement soil with or without fiber inclusion were achieved with high determination coefficients. The cement soil reinforced with basalt fiber has a better correlation of the splitting tensile strength and the compressive strength, and a better improvement in splitting tensile strength with the correlation coefficient of 0.245.
(4) The contribution of basalt fibers on the toughening mechanisms of cement soil can be explained by fiber–matrix interaction, including strong interfacial bonds, an effective bridging effect, crack deflection and branching effects, etc., which are also responsible for the improvement of overall mechanical properties of the soil-cement-fiber composites. The results of this study can provide a parameter basis for the application and popularization of cement soil in engineering.

## Figures and Tables

**Table 1:** Physical indexes of soil.

Physical and Mechanical Index	Value	Physical and Mechanical Index		Value
Natural water content (%)	36.2	Plastic limit (%)	-	22.9
Natural density (g/cm^3^)	1.8	Plasticity index	-	10.9
Void ratio	1.014	Particle size distribution (%)	0.250–0.075 mm	3.7
Saturation degree (%)	96.0		0.075–0.005 mm	85.5
Liquid limit (%)	33.8		<0.005 mm	10.8
Specific gravity	2.69	Type of soil	-	Muddy silty clay

**Table 2:** Performance parameters of alkali-resistant basalt fiber.

Performance Parameters	Value	Performance Parameters	Value
Raw material ingredient	Basalt fiber	Fiber type	Monofilament bundle
Monofilament diameter (µm)	13	Density (k/cm^3^)	2.65
Tensile strength (MPa)	≥2000	Elastic Modulus (GPa)	90~110
Ultimate elongation (%)	3.5	Acid and alkali resistance (%)	≥99
Melting point(°C)	1250	-	-

**Table 3:** Mixture design of test materials.

No.	Cement Content/%	Fiber Content/%	Fiber Length/mm	Number of Samples		
				7 Days	28 Days	90 Days
C18%	18	0	0	6	6	6
C15%	15	0	0	6	6	6
B0.2%	15	0.2	12	6	6	6
B0.4%	15	0.4	12	6	6	6
B0.6%	15	0.6	12	6	6	6
B6 mm	15	0.4	6	6	6	6
B24 mm	15	0.4	24	6	6	6

**Table 4:** Splitting tensile strength.

No.	7 Days/MPa	*C*_v_	28 Days/MPa	*C*_v_	90 Days/MPa	*C*_v_
C18%	0.24	0.03	0.36	0.03	0.60	0.08
C15%	0.20	0.01	0.28	0.03	0.45	0.05
B0.2%	0.23	0.01	0.34	0.09	0.58	0.01
B0.4%	0.29	0.04	0.42	0.08	0.66	0.05
B0.6%	0.25	0.07	0.39	0.02	0.59	0.03
B6 mm	0.27	0.02	0.37	0.01	0.56	0.05
B12 mm	0.29	0.04	0.42	0.08	0.66	0.05
B24 mm	0.25	0.08	0.38	0.05	0.54	0.07

**Table 5:** Growth ratio of tensile strength of cement soil with different basalt fiber content.

Curing Time/Day	Growth Rate Compared with Plain Cement Soil (C15%)/%		
	B0.2%	B0.4%	B0.6%
7	15.5	45.0	25.0
28	21.4	50.0	39.3
90	28.9	46.7	31.1

**Table 6:** Growth ratio of tensile strength of cement soil with different basalt fiber length.

Curing Time/Day	Growth Rate Compared with Plain Cement Soil (C15%)/%		
	B6 mm	B12 mm	B24 mm
7	35.0	45.0	25.0
28	32.1	50.0	35.7
90	24.4	46.7	20.0

**Table 7:** Unconfined compressive strength.

No.	7 Days/MPa	*C*_v_	28 Days/MPa	*C*_v_	90 Days/MPa	*C*_v_
C18%	1.08	0.01	1.52	0.04	2.28	0.07
C15%	1.03	0.03	1.29	0.05	1.90	0.02
B0.2%	1.08	0.04	1.58	0.09	2.31	0.01
B0.4%	1.16	0.03	1.67	0.03	2.43	0.07
B0.6%	1.14	0.01	1.62	0.01	2.38	0.01
B6 mm	1.12	0.08	1.54	0.03	2.40	0.04
B12 mm	1.16	0.03	1.67	0.03	2.43	0.07
B24 mm	1.09	0.01	1.51	0.07	2.18	0.07

**Table 8:** Fitting results of the relationship between tensile strength and compressive strength.

No.	Cement Content/%	Fiber Content/%	Fiber Length/mm	Correlation Coefficient, *a*	Determination Coefficient, R^2^
C18%	18	0	0	0.256	0.980
C15%	15	0	0	0.224	0.984
B0.2%	15	0.2	12	0.247	0.990
B0.4%	15	0.4	12	0.263	0.994
B0.6%	15	0.6	12	0.242	0.996
B6 mm	15	0.4	6	0.236	0.998
B24 mm	15	0.4	24	0.246	0.996
